# Is household composition associated with the presence of risk behaviors in Brazilian adolescents?

**DOI:** 10.1590/1980-549720240058

**Published:** 2024-12-13

**Authors:** Marielly Rodrigues de Souza, Ana Paula Muraro, Amanda Cristina de Souza Andrade, Márcia Gonçalves Ferreira, Paulo Rogério Melo Rodrigues

**Affiliations:** I Universidade Federal de Mato Grosso, Postgraduate Program in Collective Health – Cuiabá (MT), Brazil.; II Universidade Federal de Mato Grosso, Institute of Collective Health – Cuiabá (MT), Brazil.; III Universidade Federal de Mato Grosso, School of Nutrition – Cuiabá (MT), Brazil.

**Keywords:** Family characteristics, Risk behaviors, Life style, Health survey, Adolescents

## Abstract

**Objective:**

To analyze the association of household composition with risk behaviors in Brazilian adolescents.

**Methods:**

Cross-sectional study, with a nationally representative sample of Brazilian adolescents (n=159,245) aged 13 to 17, enrolled and regularly attending the 7th to 9th year of elementary school and the 1st to 3rd year of high school, participants in the National Survey of School Health in 2019. The risk behaviors were: insufficient physical activity, sedentary behavior, alcohol consumption, smoking, poorer diet quality, skipping breakfast and not having meals with parents/guardians. In the analyses, the sampling weights and study design were considered, stratified by the type of school (public or private) and estimated using Poisson regression models.

**Results:**

Adolescents, from public and private schools, who lived in single-parent households or where parents were absent, had a higher prevalence of alcohol consumption, smoking, poorer diet quality, skipping breakfast and not eating meals with parents/guardians, compared to those who lived with both parents. Additionally, adolescents from public schools showed a higher prevalence of sedentary behavior than those from single-parent households. Adolescents from private schools had a higher prevalence of sedentary behavior among those who lived only with their mother and a higher prevalence of insufficient physical activity among those who lived without either parent.

**Conclusion:**

Brazilian adolescents, from public and private schools, who lived in single-parent households or without parents, showed higher prevalence of risk behaviors.

## INTRODUCTION

Adolescence is a period marked by biopsychosocial changes that can predispose adolescents to health risk factors, including lifestyle-related risk behaviors, which can also affect quality of life^1,2^.

Parental support and behaviors can interfere with the development and maintenance of behaviors adopted by adolescents^3,4^ and positive parental attitudes, which promote health and quality of life and increase the likelihood of equally positive behaviors in children^5^.

The family environment can encourage the adoption of behaviors that will have a direct impact on the health and lives of adolescents, through the family socialization process^4,6^. Silva et al.^7^ found that the chance of simultaneously presenting a greater number of risk behaviors (sedentary lifestyle, low fruit consumption, and regular smoking and consumption of alcoholic beverages) was greater for adolescents who lived in single-parent or parentless households.

Thus, household composition can compose a scenario more or less prone to the adoption of different risk behaviors, which have been identified as precursors of future conditions of illness and development of several comorbidities, including chronic non-communicable diseases (NCDs)^2,7,8^. However, most studies found in the literature present isolated associations of specific behaviors or do not consider household composition as the main exposure. The objective of the present study was to analyze the association of household composition and risk behaviors in Brazilian adolescents.

## METHODS

**Figure 1:**
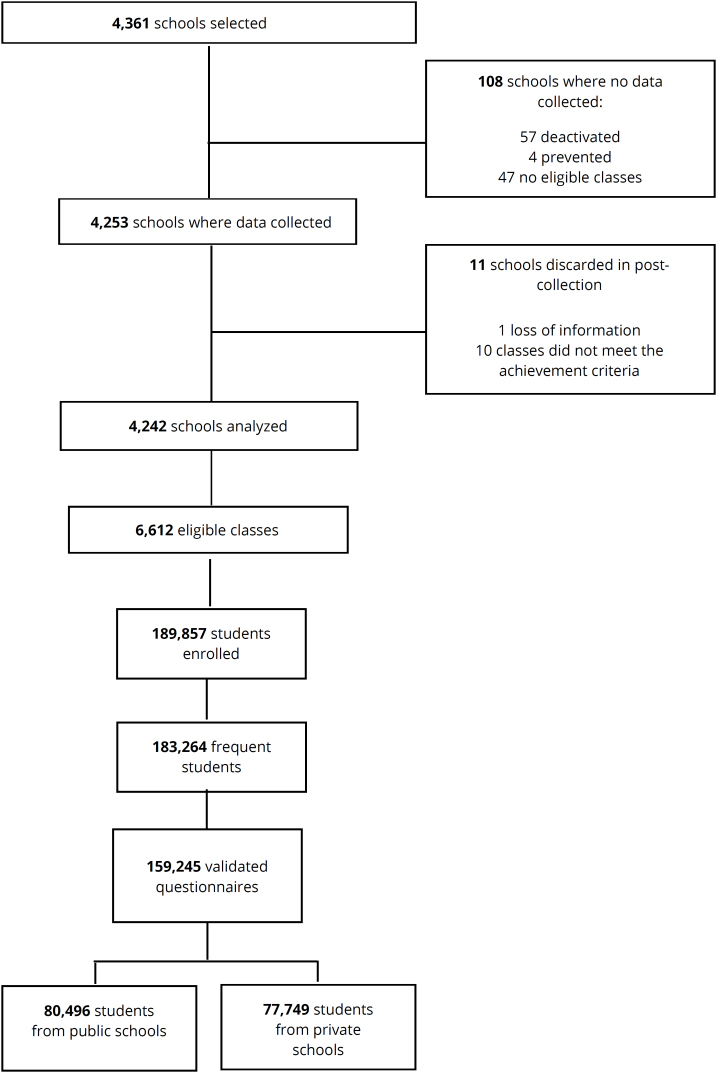
Sample flowchart of National Survey of School Health (PeNSE), 2019 Edition, Brazil^9^.

A cross-sectional, school-based study with a nationally representative sample of Brazilian adolescents, using data from the 2019 National Survey of School Health (PeNSE), which investigated students enrolled and regularly attending the 7th to 9th grades of elementary school and the 1st to 3rd grades of high school. PeNSE was conducted by the Brazilian Institute of Geography and Statistics (IBGE) in partnership with the Ministry of Health. The sample was sized to estimate population parameters representative of the population, composed of students aged 13 to 17, from public and private schools in Brazil. Sampling was done by clusters in two stages, with schools corresponding to the first stage and classes of students corresponding to the second stage^9^. More methodological details about the sampling plan can be seen in Figure 1 and obtained from an IBGE publication^9^.

Data collection was done by IBGE using structured and self-administered questionnaires made available on mobile collection devices. The database underwent a process of criticism and verification of information, to standardize the data, adjust for possible inconsistencies and create derived variables necessary for calculating the indicators^9^.

### Ethical aspects

This study was based on public data (http://www.ibge.gov.br) and the original PeNSE project was approved by the National Research Ethics Commission (registration No. 3.249.268).

### Study variables

The dependent variables were risk behaviors: insufficient physical activity, sedentary behavior, alcohol consumption, smoking, poor diet quality, skipping breakfast, and not having meals with parents/guardians.

The indicator of accumulated physical activity was obtained by adding the times of physical activity practiced in the week prior to the interview, considering eight items from three domains: commuting between home and school, physical education classes at school, and extracurricular physical activities. In the present study, adolescents who practiced <300 minutes/week were considered insufficiently active, and those who practiced ≥300 minutes/week were considered active^10^.

Sedentary behavior was assessed using the question "How many hours per day do you usually spend sitting, watching television, playing video games, using a computer, cell phone or tablet or doing other activities while sitting? (DO NOT count Saturdays, Sundays, holidays or time sitting at school)", and was categorized as less than two hours/day and equal to or greater than two hours/day^11^.

Alcohol consumption was assessed using the question "In the last 30 days (one month), on how many days did you have at least one glass or one shot of alcoholic beverage?" Adolescents who consumed at least one glass or shot of alcohol in the last 30 days were classified as consumers, and those who answered "I have never had an alcoholic beverage" or "not on any day in the last 30 days" were considered non-consumers. Responses were coded as "Yes" and "No"^12^.

Smoking was assessed by considering current cigarette consumption, obtained with the question "In the last 30 days, on how many days did you smoke cigarettes?", considering smokers as adolescents who smoked cigarettes at least one day in the last 30 days. The answers were categorized as "Yes" and "No"^13^.

To assess skipping breakfast, the question "Do you usually eat breakfast?" was used, considering the answer "No" as skipping this meal. Eating meals with parents/guardians was obtained through the question "Do you usually have lunch or dinner with your mother, father or guardian?". The answer option "No" was considered as not eating meals with parents/guardians.

Diet quality was assessed based on a nutritional score, constructed on the basis of the frequency of consumption of six markers of healthy eating (beans, legumes or vegetables and fresh fruit or fruit salad) and unhealthy eating (sweet treats, soft drinks and ultra-processed foods/fast food)^14^. The score was calculated by adding the partial scores corresponding to the weekly frequency of consumption: for healthy markers, the score ranged from 0 (did not eat) to 7 (every day); and for unhealthy markers, the score ranged from 7 (did not eat) to 0 (every day). The total score can range from 0 to 49, and the higher the score, the better the nutritional quality of the diet^14^. In the present study, the total score was categorized as <75th percentile and ≥75th percentile, with the first category being considered as the worst quality of the diet. The exposure variable was household composition, assessed by means of the questions "Do you live with your mother?" and "Do you live with your father?", both with "Yes" or "No" answer options, combined and categorized as living with both parents, living with the mother only, living with the father only, and living without either parent^7,15^.

The following covariates were considered: sex (male and female), age group (13–15 and 16–17 years) and socioeconomic stratum, characterized by the goods and services score, constructed on the basis of the following items: cell phone, computer/notebook, internet, car, motorcycle, bathroom with toilet and shower in the house and presence of a maid three or more days a week. For each item, a weight was assigned that was equivalent to the inverse of the frequency of possession or presence in the total sample evaluated. The score for each adolescent was obtained by adding the weights of the respective items, which was subsequently divided into tertiles^16,17^. The administrative dependency of the school (public and private) was used as a stratification variable.

### Statistical analysis

Statistical analyses were performed using Stata, version 16.1, considering the research design and post-stratification weights. Descriptive statistics were presented as relative frequency and 95% confidence interval (95%CI). The association of household composition with demographic and socioeconomic characteristics was estimated by non-overlapping 95%CI.

In the bivariate analysis, the association of household composition with risk behaviors was estimated by non-overlapping 95%CI. Subsequently, simple and multiple Poisson regression models were used, obtaining the crude prevalence ratio (PR_c_) and adjusted prevalence ratio (PR_adj_) and respective 95%CI. The models were stratified by the administrative dependency of the school and adjusted by sex, age group and socioeconomic stratum.

## RESULTS

Of the adolescents evaluated, 50.8% were female, 65.3% were between 13 and 15 years old, 85.5% attended public schools, 34.1% were classified in the second tertile of the socioeconomic stratum, 55.7% (95%CI 54.8–56.5) lived with both parents, 32.1% (95%CI 31.4–32.8) only with the mother, 4.8% (95%CI 4.6–5.1) only with the father and 7.4% (95%CI 7.1–7.8) without either parent. Comparing the prevalence of risk behaviors among adolescents from public schools with those from private schools, a significant difference was found for sedentary behavior (63.2 vs. 77.5%; p<0.01), alcohol consumption (25.8 vs. 22.3%; p<0.01), smoking (6.7 vs. 3.7%; p<0.01), skipping breakfast (10.0 vs. 9.4%; p=0.04), and not having meals with parents/guardians (7.3 vs. 5.1%; p<0.01) (data not shown).

Among adolescents from public schools, compared with those who lived with both parents, statistically significant differences were observed for: being insufficiently active, more prevalent among those who lived without either parent (73.2%); having sedentary behavior, more prevalent among those who lived only with their mother (66.5%) and without either parent (60.0%). Drinking alcohol and being a smoker were respectively more prevalent among adolescents who lived without either parent (34.1 and 11.5%), only with their father (30.9 and 9.4%) and only with their mother (28.3 and 8.0%).

Regarding the poorer quality of the diet, it was more prevalent among adolescents who lived only with their mother (77.2%) and without either parent (76.5%). Skipping breakfast was more prevalent among adolescents who lived only with their mother (11.4%), while not having meals with parents/guardians was most prevalent among adolescents who lived without either parent (21.0%), only with their father (11.1%) and only with their mother (8.3%) (Table 1).

**Table 1:** Characterization of risk behaviors in adolescents according to type of school and household composition. National Survey of School Health (PeNSE). Brazil, 2019 (n=159,245).

Behaviors		Public schools					Private schools				
		Total (%)	Both parents	Only with mother	Only with father	Neither parent	Total (%)	Both parents	Only with mother	Only with father	Neither parent
			% (95%CI)					% (95%CI)			
Physical activity											
	Physically active	29.4	29.6 (28.7–30.5)	29.3 (28.1–30.5)	32.2 (29.7–34.8)	26.9 (25.0–28.8)	29.8	30.4 (29.3–31.5)	28.8 (27.6–30.1)	29.3 (26.2–32.6)	26.0 (23.6–28.5)
	Insufficiently active	70.6	70.4 (69.5–71.3)	70.7 (69.5–71.3)	67.8 (65.2–70.3)	73.2 (71.2–75.0)	70.2	69.6 (68.5–70.7)	71.2 (69.8–72.4)	70.7 (70.0–73.8)	74.0 (71.5–76.4)
Sedentary behavior (hours)											
	≤2	36.8	38.5 (37.3–39.7)	33.6 (32.4–34.7)	35.5 (33.0–38.0)	40.0 (37.8–42.2)	22.5	23.1 (22.0–24.2)	20.8 (19.7–21.9)	22.8 (19.8–26.1)	21.7 (19.4–24.2)
	>2	63.2	61.5 (60.3–62.7)	66.5 (65.2–67.6)	64.5 (62.0–67.0)	60.0 (57.8–62.2)	77.5	76.9 (75.8–78.0)	79.2 (78.1–80.2)	77.2 (73.9–80.2)	78.3 (75.8–80.6)
Consumption of alcoholic beverages											
	Yes	22.3	22.5 (21.2–23.9)	28.3 (26.8–29.8)	30.9 (28.3–33.6)	34.1 (31.8–36.5)	22.3	20.4 (19.1–21.9)	25.4 (23.8–27.1)	30.8 (27.7–34.0)	29.9 (27.1–34.0)
	No	77.7	77.5 (76.2–73.2)	71.7 (70.2–71.7)	69.1 (66.4–71.7)	65.9 (63.5–68.2)	77.7	79.6 (78.2–80.9)	74.6 (72.9–76.2)	69.3 (66.0–72.3)	70.2 (67.3–72.3)
Smoking											
	Yes	6.7	4.9 (4.5–5.5)	8.0 (7.3–8.8)	9.4 (7.8–11.2)	11.5 (10.2–12.9)	3.7	3.1 (2.7–3.5)	4.7 (4.2–5.3)	6.9 (5.5–8.7)	5.4 (4.3–6.7)
	No	93.3	95.0 (94.5–95.5)	92.0 (91.2–92.8)	90.7 (88.8–92.2)	88.5 (87.1–89.8)	96.3	96.9 (96.5–97.3)	95.3 (94.7–95.8)	93.1 (91.3–94.5)	94.6 (93.3–95.7)
Diet quality											
	Better	25.4	27.4 (26.4–28.3)	22.8 (21.9–23.8)	24.2 (21.8–26.9)	23.5 (21.7–25.4)	26.0	27.3 (26.2–28.4)	22.7 (21.5–23.9)	24.0 (21.0–27.2)	25.2 (22.7–27.9)
	Worse	74.6	72.7 (71.7–73.6)	77.2 (76.2–78.1)	75.8 (73.1–78.2)	76.5 (74.6–78.3)	74.0	76.7 (71.6–73.8)	77.3 (76.1–78.5)	76.0 (72.8–79.0)	74.8 (72.1–77.3)
Breakfast											
	Regular	90.0	90.9 (90.3–91.5)	88.7 (87.9–89.4)	90.2 (88.5–91.6)	89.7 (88.5–90.8)	90.6	91.6 (91.2–92.1)	88.5 (87.9–89.3)	89.0 (86.9–90.8)	88.1 (86.1–89.9)
	Skipping	10.0	9.1 (8.5–9.7)	11.4 (10.6–12.2)	9.8 (8.4–11.5)	10.3 (9.2–11.6)	9.4	8.4 (7.9–8.9)	11.5 (10.7–12.3)	11.0 (9.2–13.1)	11.9 (10.2–13.9)
Meals with parents/guardians											
	Yes	92.7	95.7 (95.4–96.0)	91.7 (90.9–92.3)	88.9 (87.1–90.6)	79.0 (77.3–80.7)	94.9	96.5 (96.2–96.8)	92.2 (91.4–92.8)	91.9 (90.0–93.4)	83.9 (81.5–86.1)
	No	7.3	4.3 (4.0–4.6)	8.3 (7.7–9.0)	11.1 (9.4–12.9)	21.0 (19.4–22.7)	5.1	3.5 (3.2–3.8)	7.9 (7.2–8.6)	8.1 (6.7–10.0)	16.1 (13.8–18.5)

For adolescents in private schools, compared to adolescents who lived with both parents, statistically significant differences were observed for being insufficiently active: more prevalent among those who lived without either parent (74.0%); having sedentary behavior: more prevalent among adolescents who lived only with their mother (79.2%); drinking alcohol and being a smoker, respectively: more prevalent among those who lived without either parent (29.9 and 5.4%), only with the father (30.8 and 6.9%) and only with the mother (25.3 and 4.7%); poorer diet quality: more prevalent among adolescents who lived only with the mother (77.3%); skipping breakfast and not having meals with parents/guardians: more prevalent among adolescents who lived without either parent (11.9 and 16.1%), only with the father (11.0 and 8.1%) and only with the mother (11.5 and 7.9%) (Table 1).

In the adjusted analysis, in the public school stratum, adolescents who lived only with their mother had a higher prevalence of poorer diet quality (PR_adj_ =1.05), sedentary behavior (PR_adj_=1.10), skipping breakfast (PR_adj_=1.24), alcohol consumption (PR_adj_=1.27), smoking (PR_adj_=1.61) and not having meals with parents/guardians (PR_adj_ =1.82) compared to those who lived with both parents. Adolescents who lived only with their father had a higher prevalence of poorer diet quality (PR_adj_=1.04), sedentary behavior (PR_adj_ =1.05), alcohol consumption (PR_adj_ =1.31), smoking (PR_adj_=1.76) and not having meals with parents/guardians (PR_adj_ =2.52) compared to those who lived with both parents. Adolescents who lived without either parent had a higher prevalence of alcohol consumption (PR_adj_=1.32), smoking (PR_adj_=2.01), poorer diet quality (PR_adj_=1.03) and not having meals with parents/guardians (PR_adj_=4.37) compared to those who lived with both parents (Table 2).

**Table 2:** Association of household composition and risk behaviors in Brazilian adolescents from public schools. National Survey of School Health (PeNSE), 2019.

	Risk behaviors					
Household composition	Insufficient physical activity		Sedentary behavior		Consumption of alcoholic beverages	
	PR_c_ (95%CI)	PR_adj_ (95%CI)	PR_c_ (95%CI)	PR_adj_ (95%CI)	PR_c_ (95%CI)	PR_adj_ (95%CI)
Both parents	1	1	1	1	1	1
Only with mother	1.00 (0.99–1.02)	0.99 (0.97–1.01)	1.08 (1.06–1.10)*	1.10 (1.07–1.12)*	1.26 (1.20–1.32)*	1.27 (1.21–1.32)*
Only with father	0.96 (0.93–1.00)	0.98 (0.94–1.01)	1.05 (1.01–1.09)^†^	1.05 (1.01–1.09)^†^	1.37 (1.25–1.50)*	1.31 (1.20–1.4)*
Neither parent	1.04 (1.01–1.07)*	1.00 (0.98–1.03)	0.98 (0.94–1.01)	0.98 (0.95–1.02)	1.52 (1.41–1.63)*	1.32 (1.23–1.42)*

Household composition	Smoking		Poor diet quality	
	PR_c_ (95%CI)	PR_adj_ (95%CI)	PR_c_ (95%CI)	PR_adj_ (95%CI)
Both parents	1	1	1	1
Only with mother	1.61 (1.45–1.79)*	1.61 (1.45–1.79)*	1.06 (1.05–1.08)*	1.05 (1.04–1.07)*
Only with father	1.88 (1.57–2.25)*	1.76 (1.47–2.11)*	1.04 (1.01–1.08)^†^	1.04 (1.01–1.08)^†^
Neither parent	2.31 (2.05–2.61)*	2.01 (1.78–2.28)*	1.05 (1.02–1.08)*	1.03 (1.01–1.06)^†^

Household composition	Skipping breakfast		Not eating meals with parents/guardians	
	PR_c_ (IC95%)	PR_adj_ (95%CI)	PR_c_ (95%CI)	PR_adj_ (95%CI)
Both parents	1	1	1	1
Only with mother	1.25 (1.14–1.36)*	1.24 (1.13–1.35)*	1.93 (1.75–2.13)*	1.82 (1.65–2.02)*
Only with father	1.08 (0.91–1.28)	1.09 (0.93–1.29)	2.57 (2.14–3.08)*	2.52 (2.11–3.01)*
Neither parent	1.13 (0.99–1.29)	1.08 (0.95–1.23)	4.87 (4.38–5.42)*	4.37 (3.93–4.86)*

PR_c_: crude prevalence ratio; PR_adj_: adjusted prevalence ratio according to sex, age group and socioeconomic stratum; 95%CI: 95% confidence interval of *p<0.01; ^†^p<0.05.

Among adolescents in private schools, those who lived only with their mother had a higher prevalence of sedentary behavior (PR_adj_ =1.03), poorer diet quality (PR_adj_ =1.05), alcohol consumption (RP_aj_=1.23), skipping breakfast (RPaj=1.37), smoking (PR_adj_=1.52) and not having meals with parents/guardians (PR_adj_=2.10) compared to those who lived with both parents. Adolescents who lived with only their father had a higher prevalence of alcohol consumption (PR_adj_ =1.36), smoking (PR_adj_ =1.98), poorer diet quality (PR_adj_ =1.04), skipping breakfast (PR_adj_ =1.32) and not having meals with parents/guardians (PR_adj_ =2.20) compared to those who lived with both parents.

Adolescents who lived without either parent had a higher prevalence of poorer diet quality (PR_adj_=1.03), insufficient physical activity (PR_adj_=1.05), alcohol consumption (PR_adj_=1.29), smoking (PR_adj_ =1.49), skipping breakfast (PR_adj_=1.44) and not having meals with parents/guardians (PR_adj_ =4.04) compared to those who lived with both parents (Table 3).

**Table 3:** Association of household composition and risk behaviors of Brazilian adolescents in private schools. National Survey of School Health (PeNSE), 2019.

	Risk behaviors					
Household composition	Insufficient physical activity		Sedentary behavior		Consumption of alcoholic beverages	
	PR_c_ (95%CI)	PR_adj_ (95%CI)	PR_c_ (95%CI)	PR_adj_ (95%CI)	PR_c_ (95%CI)	PR_adj_ (95%CI)
Both parents	1	1	1	1	1	1
Only with mother	1.02 (1.00–1.04)*	1.00 (0.98–1.02)	1.03 (1.0–1.04)*	1.03 (1.01–1.05)^†^	1.24 (1.17–1.32)^†^	1.23 (1.16–1.29)^†^
Only with father	1.01 (0.96–1.07)	1.02 (0.97–1.07)	1.00 (0.96–1.05)	1.00 (0.96–1.05)	1.51 (1.33–1.70)^†^	1.36 (1.19–1.55)^†^
Neither parent	1.06 (1.03–1.10)^†^	1.05 (1.01–1.09)^†^	1.02 (0.98–1.05)	1.02 (0.99–1.05)	1.46 (1.32–1.61)^†^	1.29 (1.17–1.42)^†^

Household composition	Smoking		Poor diet quality	
	PR_c_ (95%CI)	PR_adj_ (95%CI)	PR_c_ (95%CI)	PR_adj_ (95%CI)
Both parents	1	1	1	1
Only with mother	1.54 (1.35–1.75)^†^	1.52 (1.32–1.74)^†^	1.06 (1.04–1.08)^†^	1.05 (1.03–1.07)^†^
Only with father	2.27 (1.77–2.91)^†^	1.98 (1.54–2.56)^†^	1.05 (1.01–1.09)*	1.04 (1.01–1.08)*
Neither parent	1.76 (1.38–2.23)^†^	1.49 (1.17–1.89)^†^	1.03 (0.99–1.07)	1.03 (1.01–1.06)*

Household composition	Skipping breakfast		Not eating meals with parents/guardians	
	PR_c_ (95%CI)	PR_adj_ (95%CI)	PR_c_ (95%CI)	PR_adj_ (95%CI)
Both parents	1	1	1	1
Only with mother	1.37 (1.26–1.48)^†^	1.37 (1.26–1.49)^†^	2.25 (2.00–2.52)^†^	2.10 (1.87–2.37)^†^
Only with father	1.31 (1.09–1.57)^†^	1.32 (1.20–1.58)^†^	2.32 (1.88–2.87)^†^	2.20 (1.79–2.72)^†^
Neither parent	1.42 (1.20–1.67)^†^	1.44 (1.22–1.71)^†^	4.60 (3.89–5.44)^†^	4.04 (3.40–4.80)^†^

PR_c_: crude prevalence ratio; PR_adj_: adjusted prevalence ratio according to sex, age group and socioeconomic stratum; 95%CI: 95% confidence interval; *p<0.05; ^†^p<0.01.

## DISCUSSION

Brazilian adolescents from public and private schools who lived in single-parent households or in households without parents were more likely to engage in risk behaviors than those who lived with both parents. Differences in the prevalence of risk behaviors were also observed according to whether the father or mother was present in the household, as well as according to the administrative dependency of the school. The results we obtained for smoking and alcohol consumption are consistent with findings reported in the literature. Othman et al.^18^ found that living with only one parent was a determinant of tobacco use among adolescents from Sudan. Bird et al.^19^ found that Mexican adolescents who lived in households with one or both biological parents absent were more likely to be smokers. Ricardo et al.^20^ found that Brazilian adolescents who did not live with both parents were more exposed to alcohol consumption and smoking. Park and Lee^21^ found that Korean adolescents from restructured families or children of single parents were more likely to smoke and drink alcohol. Among possible explanations, smoking and alcohol consumption have been reported by adolescents as a way of relieving psychological stress due to the absence of one or both parents, reinforcing the importance of the family in building specific strategies for adolescents’ mental health^22^.

In PeNSE 2019, living in single-parent households or households without either parent was associated with insufficient physical activity and sedentary behavior. Similar results were obtained by Langøy et al.^23^ and Ricardo et al.^20^, who found that, among Norwegian adolescents, living with a single parent was negatively associated with moderate to vigorous physical activity and positively associated with screen exposure for two or more hours per day^23^, and that Brazilian adolescents who did not live with both parents were more exposed to screens^20^.

Children from single-parent families report facing more barriers to practicing physical activities than those from families with both parents and receive less support from their parents because of lack of free time, workload and household responsibilities^24,25^. Other factors reported as restricting involvement in physical activity are the travel time that adolescents from single-parent families or households without either parent need to spend to visit their biological parents and the dependence on their parents to transport them to training sessions or competitions^24^. It is possible that adolescents from single-parent families have greater autonomy in making decisions about their free time, which may result in the choice of behaviors that require less effort and in the reduction of time invested in educational tasks^25^.

The home environment can also influence the eating behaviors of adolescents, as parental beliefs, attitudes and behaviors can affect the health behaviors of their children^26-28^. Thus, parental eating behaviors are associated with the eating behaviors of their children, both healthy and unhealthy^29^.

In the study Health Behaviors in School-aged Children, Lazzeri et al.^28^ found that adolescents who belonged to families with two parents were more likely to eat breakfast daily compared to those from single-parent families. Danish adolescents who belonged to other family structures (single parents or blended families) had a low frequency of eating breakfast^30^. In Brazil, Ricardo et al.^20^ found that adolescents who did not live with both parents were more exposed to irregular consumption of fruits and vegetables and regular consumption of ultra-processed foods. These results are similar to those in the present study, where it was observed that adolescents from single-parent or no-parent homes had worse diet quality and skipped breakfast compared to those who lived with both parents.

Levin et al.^31^ found that 48% of Scottish adolescents living in two-parent households ate family meals every day, and 17% of those living in single-parent households rarely or never ate family meals. This result is similar to that found in PeNSE 2019, in which adolescents from single-parent households or households without either parent had a higher prevalence of not having meals with parents/guardians. Eating family meals is associated with a lower likelihood of various risk behaviors during adolescence and with better physical and psychological health^31,32^. In addition, eating family meals has been associated with healthy eating behaviors, such as greater consumption of fruits, vegetables and dairy products and lower consumption of sweetened beverages, as well as providing moments of socializing and contact between family members^33,34^.

On the other hand, depending on its configuration, household composition may create a scenario of greater predisposition to adopting risk behaviors^7^. Parents who make up a single-parent household tend to be divided between providing for the household, having an overload of roles and meeting the demands for attention to the emotional and social needs that the adolescent requires^35^. The possible workload, both inside and outside the home, could negatively affect the time and quality of care provided to the adolescent^20,36^, and adolescents who do not live with their parents may be subject to fewer rules and limits, having a more permissive relationship on the part of their guardians^37^. Thus, families with two parents tend to show greater stability for their children when compared to reconstituted or single-parent families^38^.

It is important to emphasize that the risk behaviors assessed in this study (insufficient physical activity, smoking, poor diet quality, skipping breakfast, and not having meals with parents/guardians) were more prevalent in adolescents attending public schools. These results are similar to those of other studies^20,39^, which observed a higher prevalence of skipping breakfast^39^, alcohol consumption, smoking and irregular consumption of fruits and vegetables^20^ in students going to public schools. In Brazil, attending public schools has been associated with lower family income^40^; that is, these adolescents are more socioeconomically vulnerable and have possible repercussions on the family and social environment, since parents’ longer working hours can affect the time available to prepare meals and supervise adolescents’ risk behaviors. Identifying risk behaviors and their association with household composition becomes important, as it can help in the development of strategies to address problems and complications, as well as the prevention of NCDs^7^. Furthermore, it can contribute to the improvement of public policies to promote health, especially for single-parent families or families with other configurations, encouraging these families to have access to a healthier lifestyle.

The limitations considered in this study were the question used to assess exposure, since it refers only to living with the father and/or the mother, limiting the characterization of the different family arrangements. In the literature, there are several types of household compositions observed in contemporary times, for example, family structures composed of same-sex couples, schoolchildren who live alternately in two homes and adolescents who live with grandparents^41^. Thus, the importance of new studies that allow us to understand the most diverse family constructions is highlighted.

However, the characterization of household composition resulting from the questions in this survey has been used in other studies conducted with PeNSE^7,15,20^, thus allowing for comparability of results. Another limitation concerns the assessment of risk behaviors through self-reporting, which may lead to under- or overestimation of prevalence. However, self-reporting has been commonly used in national surveys, and PeNSE is part of the school health monitoring system that uses validated questions that are comparable to other international studies^7,42^.

This study has as its strengths the evaluation of a nationally representative sample of Brazilian adolescents from public and private schools, which assessed a wide variety of risk behaviors, which are potentially modifiable and may be the target of future interventions for health promotion. The results of this study may contribute to the scientific basis for interventions and the promotion of prevention strategies in the medium and long term, in order to contribute to desirable changes in adolescents’ risk behaviors.

It is concluded that Brazilian adolescents from public and private schools who lived in single-parent households or without either parent generally had a higher prevalence of risk behaviors compared to those who lived with both parents.

The joint promotion of health actions in the school and family context can be an effective way to seek to reduce exposure to risk behaviors in adolescence.
