# Supplementary material for: Is household composition associated with the presence of risk behaviors in Brazilian adolescents?
Source: Rev Bras Epidemiol. 2024 Dec 13;27:e240058. doi: 10.1590/1980-549720240058 (PMC11654866; doi:10.1590/1980-549720240058)
Supplement: Translation

# Translation: A composição domiciliar está associada à presença de comportamentos de risco em adolescentes brasileiros?

**DOI:** 10.1590/1980-549720240058.2

**Authors:** Marielly Rodrigues de Souza, Ana Paula Muraro, Amanda Cristina de Souza Andrade, Márcia Gonçalves Ferreira, Paulo Rogério Melo Rodrigues

**Affiliations:** I Universidade Federal de Mato Grosso, Programa de Pós-Graduação em Saúde Coletiva – Cuiabá (MT), Brasil.; II Universidade Federal de Mato Grosso, Instituto de Saúde Coletiva – Cuiabá (MT), Brasil.; III Universidade Federal de Mato Grosso, Faculdade de Nutrição – Cuiabá (MT), Brasil.

**Keywords:** Características da família, Comportamentos de risco, Estilo de vida, Inquérito de saúde, Adolescentes

## Abstract

**Objetivo:**

Analisar a associação da composição domiciliar com comportamentos de risco em adolescentes.

**Métodos:**

Estudo transversal, com amostra nacionalmente representativa de adolescentes brasileiros (n=159.245) de 13 a 17 anos, matriculados e frequentando regularmente do sétimo ao nono anos do ensino fundamental e da primeira à terceira séries do ensino médio, participantes da Pesquisa Nacional de Saúde do Escolar em 2019. Os comportamentos de risco foram prática insuficiente de atividade física, comportamento sedentário, consumo de bebida alcoólica, tabagismo, pior qualidade da dieta, omissão do café da manhã e não realização de refeições com pais/responsáveis. Nas análises, que foram estratificadas pelo tipo de escola (pública ou privada) e estimadas por modelos de regressão de Poisson, foram considerados os pesos amostrais e o desenho do estudo.

**Resultados:**

Adolescentes de escolas públicas e privadas, que moravam em domicílios monoparentais ou sem nenhum dos pais, apresentaram maior prevalência de consumo de bebida alcóolica, tabagismo, pior qualidade da dieta, omissão do café da manhã e não realização de refeições com pais/responsáveis comparados àqueles que moravam com ambos os pais. Adicionalmente, entre adolescentes de escolas públicas, aqueles que viviam em domicílios monoparentais apresentaram maior prevalência de comportamento sedentário. Dos adolescentes de escolas privadas, apresentaram maior prevalência de comportamento sedentário aqueles que moravam apenas com a mãe e maior prevalência de prática insuficiente de atividade física os que moravam sem nenhum dos pais.

**Conclusão:**

Adolescentes brasileiros, de escolas públicas e privadas, que residiam em domicílios monoparentais ou sem os pais, apresentaram maiores prevalências de comportamentos de risco.

## INTRODUÇÃO

A adolescência é um período marcado por alterações biopsicossociais que podem predispor os adolescentes a fatores de riscos à saúde, entre eles os comportamentos de risco relacionados ao estilo de vida, os quais podem afetar também a qualidade de vida^1,2^.

O apoio e os comportamentos parentais podem interferir no desenvolvimento e na manutenção dos comportamentos adotados pelos adolescentes^3,4^ e nas atitudes parentais positivas, que promovem saúde e qualidade de vida e aumentam a probabilidade de comportamentos igualmente positivos dos filhos^5^.

O ambiente familiar pode estimular a adoção de comportamentos que terão impacto direto na saúde e na vida dos adolescentes, por meio do processo de socialização familiar^4,6^. Silva et al.^7^ verificaram que a chance de apresentar simultaneamente maior número de comportamentos de risco (sedentarismo, baixo consumo de frutas e consumo regular de tabaco e de bebidas alcoólicas) foi maior para adolescentes que moravam em domicílios monoparentais ou sem os pais.

Assim, a composição domiciliar pode compor um cenário mais ou menos propenso à adoção de diferentes comportamentos de risco, os quais têm sido apontados como precursores de condições futuras de adoecimento e desenvolvimento de diversas comorbidades, entre elas as doenças crônicas não transmissíveis (DCNT)^2,7,8^. Contudo, a maioria dos estudos encontrados na literatura apresentam associações isoladas de comportamentos específicos ou não consideram a composição domiciliar como exposição principal. O objetivo do presente estudo foi analisar a associação de composição domiciliar e comportamentos de risco em adolescentes brasileiros.

## MÉTODOS

Estudo transversal de base escolar, com amostra nacionalmente representativa de adolescentes brasileiros, utilizando dados da Pesquisa Nacional de Saúde do Escolar (PeNSE), edição de 2019, que investigou escolares matriculados e que frequentaram regularmente do sétimo ao nono anos do ensino fundamental e da primeira à terceira séries do ensino médio. A PeNSE foi conduzida pelo Instituto Brasileiro de Geografia e Estatística (IBGE) em parceria com o Ministério da Saúde.

A amostra foi dimensionada para estimar parâmetros populacionais representativos da população, composta por alunos de 13 a 17 anos de idade, de escolas públicas e privadas do Brasil. A amostragem foi feita por conglomerados em dois estágios, sendo as escolas correspondentes ao primeiro estágio e as turmas de alunos ao segundo estágio^9^. Mais detalhes metodológicos sobre o plano amostral podem ser observados na Figura 1 e obtidos em publicação do IBGE^9^.

**Figura 1:** Fluxograma amostral da Pesquisa Nacional de Saúde do Escolar (PeNSE), edição 2019, Brasil.^9^

A coleta de dados foi realizada pelo IBGE por meio de questionários estruturados e autoaplicáveis disponibilizados em dispositivos móveis de coleta. A base de dados passou por processo de crítica e apuração das informações, a fim de padronizar os dados, ajustar possíveis inconsistências e criar variáveis derivadas necessárias ao cálculo dos indicadores^9^.

### Aspectos éticos

O presente estudo é baseado em dados públicos (http://www.ibge.gov.br) e o projeto original da PeNSE foi aprovado pela Comissão Nacional de Ética em Pesquisa (registro nº 3.249.268).

### Variáveis do estudo

As variáveis dependentes foram os comportamentos de risco: prática insuficiente de atividade física, comportamento sedentário, consumo de bebida alcoólica, tabagismo, pior qualidade da dieta, omissão do café da manhã e não realização das refeições com pais/responsáveis.

O indicador de atividade física acumulada foi obtido pela soma dos tempos de atividades físicas praticadas na semana anterior à da entrevista, considerando oito quesitos de três domínios: deslocamento entre casa e escola, aulas de educação física na escola e atividades físicas extraescolares. No presente estudo, foi considerado insuficientemente ativo o adolescente que praticou <300 minutos/semana e ativo aquele que praticou ≥300 minutos/semana^10^.

O comportamento sedentário foi avaliado utilizando-se a pergunta "Quantas horas por dia você costuma ficar sentado(a), assistindo à televisão, jogando videogame, usando computador, celular, tablet ou fazendo outras atividades sentado(a)? (NÃO contar sábado, domingo, feriados ou o tempo sentado na escola)", sendo categorizado em menor que duas horas/dia e igual ou maior que duas horas/dia^11^.

O consumo de bebidas alcoólicas foi avaliado por meio da pergunta "Nos últimos 30 dias (um mês), em quantos dias você tomou pelo menos um copo ou uma dose de bebida alcóolica?" Os adolescentes que consumiram pelo menos um copo ou dose de bebida nos últimos 30 dias foram classificados como consumidores e os que responderam "Nunca tomei bebida alcóolica" ou "nenhum dia nos últimos 30 dias" foram considerados como não consumidores. As respostas foram codificadas em "Sim" e "Não"^12^.

O tabagismo foi avaliado considerando o consumo atual de cigarro, obtido com a pergunta "Nos últimos 30 dias, em quantos dias você fumou cigarros?", considerando tabagistas os adolescentes que fumaram cigarro pelo menos um dia nos últimos 30 dias. As respostas foram categorizadas em "Sim" e "Não"^13^.

Para avaliar a omissão do café da manhã, utilizou-se a pergunta "Você costuma tomar o café da manhã?", considerando a resposta "Não" como omissão dessa refeição. A realização de refeições com pais/responsáveis foi obtida por meio da pergunta "Você costuma almoçar ou jantar com sua mãe, pai ou responsável?". A opção de resposta "Não" foi considerada como não realiza refeições com os pais/responsáveis.

A qualidade da dieta foi avaliada com base em um escore nutricional, construído com base na frequência de consumo de seis marcadores de alimentação saudável (feijão, legume ou verdura e frutas frescas ou salada de frutas) e não saudável (guloseimas doces, refrigerantes e alimentos ultraprocessados/fast food)^14^. O escore foi calculado somando-se os escores parciais correspondentes à frequência semanal de consumo: para os marcadores saudáveis, a pontuação variou de 0 (não consumia) a 7 (todos os dias); e para os marcadores não saudáveis, a pontuação variou de 7 (não consumia) a 0 (todos os dias). O escore total pode variar de 0 a 49, e quanto maior a pontuação, melhor a qualidade nutricional da dieta^14^. No presente estudo, o escore total foi categorizado em <percentil 75 e ≥percentil 75, sendo a primeira categoria considerada como pior qualidade da dieta.

A variável de exposição foi a composição domiciliar, avaliada por meio das questões "Você mora com sua mãe?" e "Você mora com seu pai?", ambas com opções de respostas "Sim" ou "Não", combinadas e categorizadas em morar com ambos os pais, morar apenas com a mãe, morar apenas com o pai e morar sem nenhum dos pais^7,15^.

Foram consideradas como covariáveis o sexo (masculino e feminino), a faixa etária (13–15 e 16–17 anos) e o estrato socioeconômico, caracterizado pelo escore de bens e serviços, construído com base nos itens: telefone celular, computador/notebook, internet, automóvel, moto, banheiro com vaso sanitário e chuveiro na casa e presença de empregada doméstica em três ou mais dias por semana. Para cada item, foi atribuído um peso que equivaleu ao inverso da frequência de posse ou presença no total da amostra avaliada. O escore de cada adolescente foi obtido por meio da soma dos pesos dos respectivos itens, que posteriormente foi dividido em tercis^16,17^. A dependência administrativa da escola (pública e privada) foi utilizada como variável de estratificação.

### Análise estatísticas

As análises estatísticas foram realizadas no software Stata, versão 16.1, considerando o desenho da pesquisa e os pesos pós-estratificação. A estatística descritiva foi apresentada como frequência relativa e intervalo de confiança de 95% (IC95%). A associação da composição domiciliar com as características demográficas e socioeconômicas foi estimada pela não sobreposição dos IC95%.

Na análise bivariada, a associação da composição domiciliar com os comportamentos de risco foi estimada pela não sobreposição dos IC95%. Na sequência, utilizaram-se modelos de regressão de Poisson simples e múltipla, obtendo a razão de prevalência bruta (RP_b_) e ajustada (RP_aj_) e respectivos IC95%. Os modelos foram estratificados pela dependência administrativa da escola e ajustados por sexo, faixa etária e estrato socioeconômico.

## RESULTADOS

Dos adolescentes avaliados, 50,8% eram do sexo feminino, 65,3% com idade entre 13 e 15 anos, 85,5% de escolas públicas, 34,1% foram classificados no segundo tercil do estrato socioeconômico, 55,7% (IC95% 54,8–56,5) moravam com ambos os pais, 32,1% (IC95% 31,4–32,8) apenas com a mãe, 4,8% (IC95% 4,6–5,1) apenas com o pai e 7,4% (IC95% 7,1–7,8) sem nenhum dos pais.

Comparando a prevalência dos comportamentos de risco de adolescentes de escolas públicas com aqueles de escolas privadas, verificou-se diferença significativa para comportamento sedentário (63,2 vs. 77,5%; p<0,01), consumo de bebidas alcoólicas (25,8 vs. 22,3%; p<0,01), tabagismo (6,7 vs. 3,7%; p<0,01), omissão do café da manhã (10,0 vs. 9,4%; p=0,04) e não realização de refeições com pais/responsáveis (7,3 vs. 5,1%; p<0,01) (dados não mostrados).

Entre os adolescentes de escolas públicas, comparando-se com aqueles que moravam com ambos os pais, foram observadas diferenças estatisticamente significativas para: ser insuficientemente ativo, mais prevalente entre os que moravam sem nenhum dos pais (73,2%); ter comportamento sedentário, mais prevalente entre aqueles que moravam apenas com a mãe (66,5%) e sem nenhum dos pais (60,0%). Consumir bebida alcoólica e ser tabagista, respectivamente, mais prevalentes entre adolescentes que moravam sem nenhum dos pais (34,1 e 11,5%), apenas com o pai (30,9 e 9,4%) e apenas com a mãe (28,3 e 8,0%).

Quanto à pior qualidade da dieta, foi mais prevalente entre adolescentes que moravam apenas com a mãe (77,2%) e sem nenhum dos pais (76,5%). Já omitir o café da manhã foi mais prevalente entre adolescentes que moravam apenas com a mãe (11,4%), enquanto não realizar refeições com pais/responsáveis, a maior prevalência foi constatada entre adolescentes que moravam sem nenhum dos pais (21,0%), apenas com o pai (11,1%) e apenas com a mãe (8,3%) (Tabela 1).

**Tabela 1:** Caracterização de comportamentos de risco em adolescentes, segundo o tipo de escolas e a composição domiciliar. Pesquisa Nacional de Saúde do Escolar (PeNSE). Brasil, 2019 (n=159.245).

Comportamentos		Escolas públicas					Escolas privadas				
		Total (%)	Ambos os pais	Apenas com a mãe	Apenas com o pai	Nenhum dos pais	Total (%)	Ambos os pais	Apenas com a mãe	Apenas com o pai	Nenhum dos pais
			% (IC95%)					% (IC95%)			
Atividade física											
	Fisicamente ativo	29,4	29,6 (28,7–30,5)	29,3 (28,1–30,5)	32,2 (29,7–34,8)	26,9 (25,0–28,8)	29,8	30,4 (29,3–31,5)	28,8 (27,6–30,1)	29,3 (26,2–32,6)	26,0 (23,6–28,5)
	Insuficientemente ativo	70,6	70,4 (69,5–71,3)	70,7 (69,5–71,3)	67,8 (65,2–70,3)	73,2 (71,2–75,0)	70,2	69,6 (68,5–70,7)	71,2 (69,8–72,4)	70,7 (70,0–73,8)	74,0 (71,5–76,4)
Comportamento sedentário (horas)											
	≤2	36,8	38,5 (37,3–39,7)	33,6 (32,4–34,7)	35,5 (33,0–38,0)	40,0 (37,8–42,2)	22,5	23,1 (22,0–24,2)	20,8 (19,7–21,9)	22,8 (19,8–26,1)	21,7 (19,4–24,2)
	>2	63,2	61,5 (60,3–62,7)	66,5 (65,2–67,6)	64,5 (62,0–67,0)	60,0 (57,8–62,2)	77,5	76,9 (75,8–78,0)	79,2 (78,1–80,2)	77,2 (73,9–80,2)	78,3 (75,8–80,6)
Consumo de bebida alcoólica											
	Sim	22,3	22,5 (21,2–23,9)	28,3 (26,8–29,8)	30,9 (28,3–33,6)	34,1 (31,8–36,5)	22,3	20,4 (19,1–21,9)	25,4 (23,8–27,1)	30,8 (27,7–34,0)	29,9 (27,1–34,0)
	Não	77,7	77,5 (76,2–73,2)	71,7 (70,2–71,7)	69,1 (66,4–71,7)	65,9 (63,5–68,2)	77,7	79,6 (78,2–80,9)	74,6 (72,9–76,2)	69,3 (66,0–72,3)	70,2 (67,3–72,3)
Tabagismo											
	Sim	6,7	4,9 (4,5–5,5)	8,0 (7,3–8,8)	9,4 (7,8–11,2)	11,5 (10,2–12,9)	3,7	3,1 (2,7–3,5)	4,7 (4,2–5,3)	6,9 (5,5–8,7)	5,4 (4,3–6,7)
	Não	93,3	95,0 (94,5–95,5)	92,0 (91,2–92,8)	90,7 (88,8–92,2)	88,5 (87,1–89,8)	96,3	96,9 (96,5–97,3)	95,3 (94,7–95,8)	93,1 (91,3–94,5)	94,6 (93,3–95,7)
Qualidade da dieta											
	Melhor	25,4	27,4 (26,4–28,3)	22,8 (21,9–23,8)	24,2 (21,8–26,9)	23,5 (21,7–25,4)	26,0	27,3 (26,2–28,4)	22,7 (21,5–23,9)	24,0 (21,0–27,2)	25,2 (22,7–27,9)
	Pior	74,6	72,7 (71,7–73,6)	77,2 (76,2–78,1)	75,8 (73,1–78,2)	76,5 (74,6–78,3)	74,0	76,7 (71,6–73,8)	77,3 (76,1–78,5)	76,0 (72,8–79,0)	74,8 (72,1–77,3)
Café da manhã											
	Regular	90,0	90,9 (90,3–91,5)	88,7 (87,9–89,4)	90,2 (88,5–91,6)	89,7 (88,5–90,8)	90,6	91,6 (91,2–92,1)	88,5 (87,9–89,3)	89,0 (86,9–90,8)	88,1 (86,1–89,9)
	Omissão	10,0	9,1 (8,5–9,7)	11,4 (10,6–12,2)	9,8 (8,4–11,5)	10,3 (9,2–11,6)	9,4	8,4 (7,9–8,9)	11,5 (10,7–12,3)	11,0 (9,2–13,1)	11,9 (10,2–13,9)
Refeições com pais/responsáveis											
	Sim	92,7	95,7 (95,4–96,0)	91,7 (90,9–92,3)	88,9 (87,1–90,6)	79,0 (77,3–80,7)	94,9	96,5 (96,2–96,8)	92,2 (91,4–92,8)	91,9 (90,0–93,4)	83,9 (81,5–86,1)
	Não	7,3	4,3 (4,0–4,6)	8,3 (7,7–9,0)	11,1 (9,4–12,9)	21,0 (19,4–22,7)	5,1	3,5 (3,2–3,8)	7,9 (7,2–8,6)	8,1 (6,7–10,0)	16,1 (13,8–18,5)

Para adolescentes de escolas privadas, comparando-se com adolescentes que moravam com ambos os pais, foram observadas diferenças estatisticamente significativas para ser insuficientemente ativo: mais prevalente entre aqueles que moravam sem nenhum dos pais (74,0%); ter comportamento sedentário: mais prevalente entre adolescentes que moravam apenas com a mãe (79,2%); consumir bebida alcoólica e ser tabagista, respectivamente: mais prevalentes entre os que moravam sem nenhum dos pais (29,9 e 5,4%), apenas com o pai (30,8 e 6,9%) e apenas com a mãe (25,3 e 4,7%); pior qualidade da dieta: mais prevalente entre adolescentes que moravam apenas com a mãe (77,3%); omitir o café da manhã e não realizar refeições com pais/responsáveis: mais prevalentes entre adolescentes que moravam sem nenhum dos pais (11,9 e 16,1%), apenas com o pai (11,0 e 8,1%) e apenas com a mãe (11,5 e 7,9%) (Tabela 1).

Na análise ajustada, no estrato de escolas públicas, adolescentes que moravam apenas com a mãe apresentaram maior prevalência de pior qualidade da dieta (RP_aj_=1,05), comportamento sedentário (RP_aj_=1,10), omissão do café da manhã (RP_aj_=1,24), consumo de bebida alcóolica (RP_aj_=1,27), tabagismo (RP_aj_=1,61) e não realização de refeições com pais/responsáveis (RP_aj_=1,82) comparados àqueles que moravam com ambos os pais. Adolescentes que moravam apenas com o pai apresentaram maior prevalência de pior qualidade da dieta (RP_aj_=1,04), comportamento sedentário (RP_aj_=1,05), consumo de bebida alcoólica (RP_aj_=1,31), tabagismo (RP_aj_=1,76) e não realização de refeições com pais/responsáveis (RP_aj_=2,52) comparados aos que moravam com ambos os pais.

Adolescentes que moravam sem nenhum dos pais apresentaram maior prevalência de consumo de bebida alcóolica (RP_aj_=1,32), tabagismo (RP_aj_=2,01), pior qualidade da dieta (RP_aj_=1,03) e não realização de refeições com pais/responsáveis (RP_aj_=4,37) comparados àqueles que moravam com ambos os pais (Tabela 2).

**Tabela 2:** Associação de composição domiciliar e comportamentos de risco em adolescentes brasileiros de escolas públicas. Pesquisa Nacional de Saúde do Escolar (PeNSE), 2019.

	Comportamentos de risco					
Composição domiciliar	Prática insuficiente de atividade física		Comportamento sedentário		Consumo de bebida alcoólica	
	RPb (IC95%)	RPaj (IC95%)	RPb (IC95%)	RPaj (IC95%)	RPb (IC95%)	RPaj (IC95%)
Ambos os pais	1	1	1	1	1	1
Apenas com mãe	1,00 (0,99–1,02)	0,99 (0,97–1,01)	1,08 (1,06–1,10)*	1,10 (1,07–1,12)*	1,26 (1,20–1,32)*	1,27 (1,21–1,32)*
Apenas com pai	0,96 (0,93–1,00)	0,98 (0,94–1,01)	1,05 (1,01–1,09)†	1,05 (1,01–1,09)†	1,37 (1,25–1,50)*	1,31 (1,20–1,4)*
Nenhum dos pais	1,04 (1,01–1,07)*	1,00 (0,98–1,03)	0,98 (0,94–1,01)	0,98 (0,95–1,02)	1,52 (1,41–1,63)*	1,32 (1,23–1,42)*

Composição domiciliar	Tabagismo		Pior qualidade da dieta	
	RPb (IC95%)		RPaj (IC95%)		RPb (IC95%)		RPaj (IC95%)	
Ambos os pais	1	1	1	1
Apenas com mãe	1,61 (1,45–1,79)*	1,61 (1,45–1,79)*	1,06 (1,05–1,08)*	1,05 (1,04–1,07)*
Apenas com pai	1,88 (1,57–2,25)*	1,76 (1,47–2,11)*	1,04 (1,01–1,08)†	1,04 (1,01–1,08)†
Nenhum dos pais	2,31 (2,05–2,61)*	2,01 (1,78–2,28)*	1,05 (1,02–1,08)*	1,03 (1,01–1,06)†

Composição domiciliar	Omissão do café da manhã		Não realização de refeições com pais/responsáveis	
	RPb (IC95%)	RPaj (IC95%)	RPb (IC95%)	RPaj (IC95%)
Ambos os pais	1	1	1	1
Apenas com mãe	1,25 (1,14–1,36)*	1,24 (1,13–1,35)*	1,93 (1,75–2,13)*	1,82 (1,65–2,02)*
Apenas com pai	1,08 (0,91–1,28)	1,09 (0,93–1,29)	2,57 (2,14–3,08)*	2,52 (2,11–3,01)*
Nenhum dos pais	1,13 (0,99–1,29)	1,08 (0,95–1,23)	4,87 (4,38–5,42)*	4,37 (3,93–4,86)*

RP_b_: razão de prevalência bruta; RP_aj_: razão de prevalência ajustada por sexo, faixa etária e estrato socioeconômico; IC95%: intervalo de confiança de 95%. *p<0,01; ^†^p<0,05.

Entre os adolescentes de escolas privadas, aqueles que moravam apenas com a mãe apresentaram maior prevalência de comportamento sedentário (RP_aj_=1,03), pior qualidade da dieta (RP_aj_=1,05), consumo de bebida alcóolica (RP_aj_=1,23), omissão do café da manhã (RP_aj_=1,37), tabagismo (RP_aj_=1,52) e não realização de refeições com pais/responsáveis (RP_aj_=2,10) comparados àqueles que moravam com ambos os pais. Adolescentes que moravam apenas com o pai apresentaram maior prevalência de consumo de bebida alcóolica (RP_aj_=1,36), tabagismo (RP_aj_=1,98), pior qualidade da dieta (RP_aj_=1,04), omissão do café da manhã (RP_aj_=1,32) e não realização de refeições com pais/responsáveis (RP_aj_=2,20) comparados aos que moravam com ambos os pais.

Adolescentes que moravam sem nenhum dos pais apresentaram maior prevalência de pior qualidade da dieta (RP_aj_=1,03), atividade física insuficiente (RP_aj_=1,05), consumo de bebida alcóolica (RP_aj_=1,29), tabagismo (RP_aj_=1,49), omissão do café da manhã (RP_aj_=1,44) e não realização de refeições com pais/responsáveis (RP_aj_=4,04) comparados àqueles que moravam com ambos os pais (Tabela 3).

**Tabela 3:** Associação de composição domiciliar e comportamentos de risco em adolescentes brasileiros de escolas privadas. Pesquisa Nacional de Saúde do Escolar (PeNSE), 2019.

	Comportamentos de risco					
Composição domiciliar	Prática insuficiente de atividade física		Comportamento sedentário		Consumo de bebida alcoólica	
	RPb (IC95%)	RPaj (IC95%)	RPb (IC95%)	RPaj (IC95%)	RPb (IC95%)	RPaj (IC95%)
Ambos os pais	1	1	1	1	1	1
Apenas com mãe	1,02 (1,00–1,04)*	1,00 (0,98–1,02)	1,03 (1,0–1,04)*	1,03 (1,01–1,05)†	1,24 (1,17–1,32)†	1,23 (1,16–1,29)†
Apenas com pai	1,01 (0,96–1,07)	1,02 (0,97–1,07)	1,00 (0,96–1,05)	1,00 (0,96–1,05)	1,51 (1,33–1,70)†	1,36 (1,19–1,55)†
Nenhum dos pais	1,06 (1,03–1,10)†	1,05 (1,01–1,09)†	1,02 (0,98–1,05)	1,02 (0,99–1,05)	1,46 (1,32–1,61)†	1,29 (1,17–1,42)†

Composição domiciliar	Tabagismo		Pior qualidade da dieta	
	RPb (IC95%)	RPaj (IC95%)	RPb (IC95%)	RPaj (IC95%)
Ambos os pais	1	1	1	1
Apenas com mãe	1,54 (1,35–1,75)†	1,52 (1,32–1,74)†	1,06 (1,04–1,08)†	1,05 (1,03–1,07)†
Apenas com pai	2,27 (1,77–2,91)†	1,98 (1,54–2,56)†	1,05 (1,01–1,09)*	1,04 (1,01–1,08)*
Nenhum dos pais	1,76 (1,38–2,23)†	1,49 (1,17–1,89)†	1,03 (0,99–1,07)	1,03 (1,01–1,06)*

Composição domiciliar	Omissão do café da manhã		Não realização de refeições com pais/responsáveis	
	RPb (IC95%)	RPaj (IC95%)	RPb (IC95%)	RPaj (IC95%)
Ambos os pais	1	1	1	1
Apenas com mãe	1,37 (1,26–1,48)†	1,37 (1,26–1,49)†	2,25 (2,00–2,52)†	2,10 (1,87–2,37)†
Apenas com pai	1,31 (1,09–1,57)†	1,32 (1,20–1,58)†	2,32 (1,88–2,87)†	2,20 (1,79–2,72)†
Nenhum dos pais	1,42 (1,20–1,67)†	1,44 (1,22–1,71)†	4,60 (3,89–5,44)†	4,04 (3,40–4,80)†

RP_b_: razão de prevalência bruta; RP_aj_: razão de prevalência ajustada por sexo, faixa etária e estrato socioeconômico. IC95%: intervalo de confiança de 95%; *p<0,05; †p<0,01.

## DISCUSSÃO

Adolescentes brasileiros, de escolas públicas e privadas, que moravam em domicílios monoparentais ou sem a presença dos pais apresentaram maior frequência de comportamentos de risco comparados àqueles que moravam com ambos os pais. Observou-se também diferenças nas prevalências nos comportamentos de risco de acordo com a presença paterna ou materna no domicílio, assim como em função da dependência administrativa da escola.

Os resultados observados no presente estudo para tabagismo e consumo de bebida alcoólica são consistentes com os observados na literatura. Othman et al.^18^ constataram, entre adolescentes do Sudão, que morar com apenas um dos pais foi determinante para o uso de tabaco. Bird et al.^19^ verificaram que adolescentes mexicanos que moravam em domicílios com ausência de um ou ambos os pais biológicos tiveram maior probabilidade de serem fumantes. Ricardo et al.^20^ observaram que adolescentes brasileiros que não moravam com ambos os pais estavam mais expostos ao consumo de bebidas alcoólicas e tabagismo. Park e Lee^21^ constataram que adolescentes coreanos de famílias reestruturadas ou filhos de pais solteiros tinham maiores chances de fumar e consumir bebidas alcoólicas. Entre possíveis explicações, o tabagismo e o consumo de bebidas alcoólicas têm sido relatados por adolescentes como forma de alívio dos estresses psicológicos pela ausência de um ou ambos os pais, reforçando a importância da família na construção de estratégias específicas para a saúde mental dos adolescentes^22^.

Na PeNSE 2019, morar em domicílios monoparentais ou sem nenhum dos pais foi associado com atividade física insuficiente e comportamento sedentário. Resultados semelhantes foram encontrados por Langøy et al.^23^ e Ricardo et al.^20^, que verificaram, entre adolescentes noruegueses, que morar com um dos pais solteiros foi negativamente associado à prática de atividade física moderada a vigorosa e positivamente associado à exposição à tela por duas horas ou mais por dia^23^ e que adolescentes brasileiros que não moravam com ambos os pais estavam mais expostos às telas^20^.

Crianças de famílias monoparentais relatam enfrentar mais barreiras para a prática de atividades físicas do que aquelas de famílias com ambos os pais e recebem menos apoio dos pais pela falta de tempo livre, carga de trabalho e responsabilidades domésticas^24,25^. Outros fatores relatados como restritores do envolvimento em atividade física são o tempo de viagem que adolescentes de famílias monoparentais ou sem nenhum dos pais precisam disponibilizar para visitar os pais biológicos e a dependência dos pais para transportá-los a treinos ou competições^24^. É possível que adolescentes de famílias monoparentais tenham maior autonomia na tomada de decisões sobre seu tempo livre, podendo resultar na escolha de comportamentos que exijam menos esforço e na redução do tempo investido em tarefas educativas^25^.

O ambiente domiciliar também pode influenciar os comportamentos alimentares dos adolescentes, pois crenças, atitudes e comportamentos parentais podem afetar os comportamentos de saúde dos filhos^26 28^. Assim, os comportamentos alimentares dos pais estão associados aos comportamentos alimentares dos filhos, tanto os saudáveis quanto não saudáveis^29^.

No estudo Health Behaviours in School-aged Children, Lazzeri et al.^28^ constataram que adolescentes que pertenciam a famílias com ambos os pais tinham maior probabilidade de consumir o café da manhã diariamente comparadas àqueles de famílias monoparentais. Adolescentes dinamarqueses que pertenciam a outras estruturas familiares (pai ou mãe solteiros ou família reconstituídas) tiveram baixa frequência de consumo de café da manhã^30^. No Brasil, Ricardo et al.^20^ constataram que adolescentes que não moravam com ambos os pais estavam mais expostos ao consumo irregular de frutas e hortaliças e consumo regular de alimentos ultraprocessados. Esses resultados se assemelham aos encontrados no presente estudo, que observou que adolescentes de domicílios monoparentais ou sem nenhum dos pais apresentaram pior qualidade da dieta e a omissão do café da manhã comparados àqueles que moravam com ambos os pais.

Levin et al.^31^ verificaram que 48% dos adolescentes escoceses que viviam em famílias compostas por ambos os pais faziam refeição em família todos os dias e 17% dos que viviam em famílias monoparentais quase nunca ou nunca faziam refeição em família. Resultado semelhante ao verificado na PeNSE 2019, em que adolescentes de domicílios monoparentais ou sem nenhum dos pais apresentaram maior prevalência de não realização de refeições com pais/responsáveis. A realização de refeições em família está associada à menor probabilidade de diversos comportamentos de risco durante a adolescência e à melhor saúde física e psicológica^31,32^. Além disso, realizar refeições em família tem sido associado a comportamentos alimentares saudáveis, como maior consumo de frutas, vegetais e produtos lácteos e menor consumo de bebidas adoçadas, assim como proporciona momentos de convívio e contato entre os familiares^33,34^.

Por outro lado, dependendo da sua configuração, a composição domiciliar pode compor um cenário de maior predisposição à adoção de comportamentos de risco^7^. Pais que compõem o núcleo monoparental tendem a estar divididos entre sustento da casa, sobrecarga de papéis e suprimento da demanda de atenção às necessidades emocionais e sociais que o adolescente requer^35^. A possível carga de trabalho, fora e dentro de casa, poderia afetar negativamente o tempo e a qualidade do cuidado oferecido ao adolescente^20,36^ e adolescentes que não moram com os pais podem estar sujeitos a ter menos regras e limites, tendo relação mais permissiva por parte de seus responsáveis^37^. Assim, famílias com ambos os pais tendem a apresentar contextos de maior estabilidade para os filhos quando comparadas às famílias reconstituídas ou monoparentais^38^.

É importante ressaltar que os comportamentos de risco avaliados neste estudo (atividade física insuficiente, tabagismo, pior qualidade da dieta, omissão do café da manhã e não realização de refeições com pais/responsáveis) foram mais prevalentes em adolescentes de escolas públicas. Esses resultados se assemelham aos de outros estudos^20,39^, que observaram maior prevalência de omissão do café da manhã^39^, consumo de bebida alcoólica, tabagismo e consumo irregular de frutas e hortaliças^20^ em estudantes de escolas públicas. No Brasil, frequentar a escola pública tem sido associado a menor renda familiar^40^, ou seja, são adolescentes com maior vulnerabilidade socioeconômica e possíveis reflexos no ambiente familiar e social, uma vez que a maior jornada de trabalho dos pais pode afetar o tempo disponível para preparar refeições e supervisionar os comportamentos de risco dos adolescentes.

Identificar os comportamentos de risco e sua associação com a composição domiciliar se torna importante, pois pode auxiliar no desenvolvimento de estratégias para o enfrentamento dos problemas e agravos, assim como a prevenção de DCNTs^7^. Além disso, pode contribuir para o aprimoramento de políticas públicas de promoção à saúde, principalmente para famílias monoparentais ou com outras configurações, estimulando o acesso dessas famílias a um estilo de vida mais saudável.

As limitações consideradas neste estudo foram a pergunta utilizada para avaliar a exposição, pois refere-se apenas a morar com o pai e/ou com a mãe, limitando a caracterização dos diferentes arranjos familiares. Na literatura, existem diversos tipos de composições domiciliares observados na contemporaneidade, por exemplo, estruturas familiares compostas por casais do mesmo sexo, escolares que moram alternadamente em dois lares e adolescentes que moram com avós^41^. Assim, destaca-se a importância de novos estudos que permitam compreender as mais diversas construções familiares.

No entanto, a caracterização da composição domiciliar resultante das perguntas da presente pesquisa tem sido utilizada em outros estudos realizados com a PeNSE^7,15,20^, permitindo assim a comparabilidade dos resultados. Outra limitação diz respeito à avaliação dos comportamentos de risco por meio do autorrelato, o que pode levar à sub ou superestimação de prevalências. Contudo, o autorrelato tem sido comumente usado em inquéritos nacionais, e a PeNSE integra o sistema de monitoramento da saúde do escolar que utiliza perguntas validadas e comparáveis a outros estudos internacionais^7,42^.

Esse estudo apresenta como pontos fortes a avaliação de amostra nacionalmente representativa de adolescentes brasileiros de escolas públicas e privadas, que avaliou ampla variedade de comportamentos de risco, que são potencialmente modificáveis e podem ser alvo de futuras intervenções para promoção da saúde. Os resultados do presente estudo podem contribuir para o embasamento científico visando a intervenções e à promoção de estratégias de prevenção em médio e longo prazos, a fim de contribuir para mudanças desejáveis nos comportamentos de risco dos adolescentes.

Concluise que adolescentes brasileiros de escolas públicas e privadas que moravam em domicílios monoparentais ou sem nenhum dos pais apresentaram, de modo geral, maior prevalência de comportamentos de risco comparados àqueles que moravam com ambos os pais.

A promoção de ações de saúde no contexto escolar e familiar, de forma conjunta, pode ser uma maneira eficiente de buscar reduzir a exposição a comportamentos de risco na adolescência.
